# Three-Month Effect of Silver Diamine Fluoride (SDF) in Salivary Levels of Streptococcus Mutans in Children. An Exploratory Trial

**DOI:** 10.3290/j.ohpd.a43360

**Published:** 2020-07-04

**Authors:** Marta Diogo Garrastazu, Ingrid Fernandes Mathias-Santamaria, Rafael Santos Rocha, Michele Baffi Diniz, Taciana Marco Ferraz Caneppele, Eduardo Bresciani

**Affiliations:** a Assistant Professor, West of Santa Catarina University (Unoesc), Joaçaba, SC, Brazil. Responsible for the design and the clinical phase of this project, and writing of the manuscript.; b Assistant Researcher, Department of Diagnosis and Surgery, Institute of Science and Technology, São José dos Campos, SP, Brazil. Discussion and manuscript writing.; c PhD Student, GAPEC – Academic Group of Clinical Research, Department of Restorative Dentistry, São Paulo State University (Unesp), Institute of Science and Technology, São José dos Campos, SP, Brazil. Discussion and manuscript writing.; d Assistant Professor, Pediatric Dentistry, Institute of Dentistry, Cruzeiro do Sul University (UNICSUL), São Paulo, SP, Brazil. Discussion and manuscript writing.; e Associate Professor, GAPEC – Academic Group of Clinical Research, Department of Restorative Dentistry, São Paulo State University (Unesp), Institute of Science and Technology, São José dos Campos, SP, Brazil. Discussion and manuscript writing.; f Associate Professor, GAPEC - Academic Group of Clinical Research, Department of Restorative Dentistry, São Paulo State University (Unesp), Institute of Science and Technology, São José dos Campos, SP, Brazil. Responsible for the design, clinical phase, statistical analysis and manuscript writing.

**Keywords:** dental caries, silver diamine fluoride, Streptococcus mutans

## Abstract

**Purpose:**

The aim of this exploratory trial was to compare the 3-month effect of two antimicrobials on the salivary levels of *Streptococcus mutans* (SM) in children.

**Materials and Methods:**

Ninety school children aged 6–10 years participated. They were divided into two groups according to treatment used: 1% chlorhexidine gel (CHX) or 30% silver diamine fluoride (SDF). Saliva for SM colony forming unit (CFU)/ml counting was harvested in four periods: baseline (prior to antimicrobials); P1 (24 h after antimicrobial therapy); P30 (30 days after antimicrobial therapy); and P90 (90 days after antimicrobial therapy). CFU/ml data was submitted to repeated measures by analysis of variance (ANOVA).

**Results:**

Only the time factor influenced the results (p <0.001), with a reduction of SM for all evaluated periods in comparison to the baseline. No influence of antimicrobials or interactions of factors were detected (p >0.05). P30 presented the lowest levels of SM and at P90, SM levels were similar to P1 but still lower than the baseline observations. SDF and CHX presented a similar effect on SM within each period of evaluation (p = 0.65).

**Conclusion:**

It was concluded that 30% SDF presents similar antimicrobial effects as 1% CHX over time. SDF might be used as an adjunctive therapy for controlling dental caries in children.

With the current knowledge in cariology regarding dental caries, many strategies have been invested in to control initial lesions of this disease.^2,17^ Data from the World Health Organization show that the prevalence of caries has declined over the decades.^24^ Nonetheless, it is still a problem that negatively affects the quality of life of a large part of the world’s population.^11,23,24^ It is known that dental caries is the most prevalent disease in the world,^23^ being five times more prevalent than asthma in North American children.^26^

The decline in the disease is due to the introduction of fluoridation of the water supply and the widespread use of fluoride toothpastes, as well as the increase of dental health education programmes and the decentralisation of public health services.^20^ However, there are areas of exceptions to this trend that require additional preventive approaches.^24^ It is reported that socioeconomically less favoured populations have a higher risk of lesions and/or experience of caries, and this association is even stronger in developed countries.^23^ Is estimated that untreated caries in permanent teeth affect 2.4 billion people around the world, and 621 million people have untreated caries in deciduous teeth.^11^

A current employed protocol for dental caries prevention is based on employing chlorhexidine. There is an association between high levels of microorganisms present in the saliva of patients with a high risk of caries disease.^4,12,14^ Therefore, chlorhexidine has an important role because it is an antimicrobial agent well tolerated by patients. It presents low toxicity, it is easy to use, has ample time for action, and its retention mechanism in the mucosa facilitates the extension of its activities in the oral environment. For those reasons, it has been considered the gold standard in research, and considered to be the most effective oral antimicrobial agent.^15,16^

It seems the previously mentioned preventive treatment of dental caries does not solve the whole problem. Thus, within that context, silver diamine fluoride (SDF) is another treatment option that has shown positive effects in the control of initial enamel caries lesions.^9,17,22^ It was introduced in the 1980 s in Japan and its formulation (Ag(NH_3_)_2_F) combines the already known properties of fluoride in tooth structure with silver metal. SDF increases the surface hardness of the enamel because it reduces mineral loss^21,25^ by forming fluorohydroxyapatite.^19^ It is also safe, effective and inexpensive.^9^ One of the reported disadvantages is the tooth staining when using this substance. Although it is reported that the chemical compost could stain the dental structure, this phenomenon was perceived by only around 7% of the parents of schoolchildren in a prospective controlled clinical trial.^5^ It has also been reported that application of potassium iodide solution minimises tooth staining, without reducing SDF’s effectiveness.^13^

Studies on SDF indicate that silver has the ability to interact with bacteria, causing bacterial death and also acts as an inhibitor of the dental biofilm formation.^29^ However, it is not known if there is an antimicrobial long-term effect of this medicine. The association of possible antimicrobial effect with the known remineralisation characteristics would strongly endorse the use of such medication.

Therefore, due to growing prevalence of caries in certain groups and the need of specific preventive measures for them, the aim of this study was to evaluate the longitudinal effect of SDF on salivary levels of *Streptococcus mutans* (SM) in the cavity oral compared with chlorhexidine, by means of an exploratory study.

## Material and Methods

### Ethics

The local institutional Review Board (UNOESC/HUST) approved the present study under protocol #105.657. Parents/guardians granted written informed consent for children participating in the research.

### Study Design

This prospective exploratory clinical study evaluated the longitudinal efficacy of 30% SDF in reducing the number of SM in high caries risk children, in comparison to 1% chlorhexidine gluconate (CHX) gel therapy.

A sample size calculation for the study was performed. Considering the reported standard deviation in salivary *S. mutans*, while assessing a 4-week influence of 1% chlorhexidine gel (0.8 log10 CFU/ml),^27^ and assuming a statistical significance level at 5%, power at 80%, and equivalence limit (d) at 0.7, a total sample size of 29 individuals per group was necessary to detect differences greater than 0.7 log10 CFU/ml within the groups.

Informed consent was distributed to parents/guardians of 1152 schoolchildren and 300 were returned. The study enrolled 90 schoolchildren, ages from 6 to 10 years, from the Joaçaba municipality in Santa Catarina, Brazil, following the inclusion and exclusion criteria previously determined.

Inclusion criteria consisted of: healthy children with no systemic condition; ages 6 to 10 years; children presenting at least one deciduous or permanent tooth scored as 1B, 2W, 2B, 3, or 4 according to International Caries Detection and Assessment System (ICDAS) assessment; and children with SM counting equal or greater than 10^6^ CFU/ml. Exclusion criteria consisted of children with the following: special care needs; an orthodontic appliance; under antibiotic therapy within the last 3 months prior to the study; with a tooth scored as 5 (enamel cavitation) or 6 (cavitation into 1/3 of dentine) from ICDAS system; having cavitated interproximal lesions, dental hypoplasia, tooth pain, presence of fistula or swelling; denied participation in the study; with no written informed consent granted; and with inclusion requirements not met.

Children not participating in the study due to caries lesions meeting the exclusion criteria or their consequences (pain and fistula) were referred to the Childs Clinic of the local university for dental treatment.

### ICDAS Calibration for Enrolment

For children enrolled in the study, the examiner was calibrated according to the ICDAS scoring system at two stages. Firstly, the examiner took an online course, an e-learning programme, from the ICDAS foundation at www.icdas.org. Secondly, the examiner was calibrated clinically, with resulting interexaminer kappa at 0.8. Codes 1B to 4 guarantee subject inclusion. Scores were distributed within groups in frequency and percentage, for permanent and deciduous teeth, as follows: CHX, score 1B = 21 (3.1%) and 18 (1.5%), score 2W = 5 (0.5%) and 10 (1.5%), score 2B = 4 (0.6%) and 3 (0.5%), score 3 = 7 (1.0%) and 27 (4.0%), and score 4 = 8 (1.2%) and 48 (7.1%); SDF, score 1B = 7 (0.8%) and 29 (3.3%), score 2W = 14 (1.6%) and 45 (5.2%), score 2B = 8 (0.9%) and 8 (0.9%), score 3 = 8 (0.9%) and 26 (3.0%), and score 4 = 3 (0.35%) and 67 (7.7%).

### Antimicrobial Treatments

Randomisation was performed by random allocation by treatment option using a sealed envelope. Treatment options, according to the number of enrolled patients, were placed inside an opaque envelope and on the first day of antimicrobial therapy the treatment was revealed to the operator. The patients, parents and evaluators did not know which treatment was performed on whom.

Antimicrobials were applied after supervised brushing. In the test group, patients received a single application of 30% SDF (Cariestop, Biodinâmica, Brazil) for 3 min with a cotton swab. In the control group, patients received one application of 1% chlorhexidine gel (Pharmacy Manipulation Formularium, Joaçaba, Brazil) with a cotton swab for 1 min. In both groups, patients were instructed not to eat or drink for 1 h.

### Microbiological Assessment

Patients received saliva stimulation with sour-flavoured diet bubble gum (Trident, São Paulo, Brazil). The first minute of stimulated saliva was discarded. During the following 2 min, saliva was harvested and conditioned in a bacteriological oven prior to evaluation. Saliva was harvested solely during the morning period.

Detection and quantification of SM were obtained by the spread plate culture technique,^28^ in mitis salivarius agar (MSB), supplemented with 1% potassium tellurite, 0.2 units/ml bacitracin, and 15% sucrose. MSB plates were visually assessed and the CFU counted by a blind evaluator.

Saliva was harvested at four times: baseline (initial harvesting) – performed prior to any antimicrobial treatment and used as an inclusion parameter (schoolchildren presenting 10^6^ CFU/ml or more were considered for the study); P1 – performed 24 h after the antimicrobial therapy; P30 – performed 30 days after the antimicrobial therapy; and P90 – performed 90 days after the antimicrobial therapy.

### Statistical Analysis

All data were transformed into log^10^ base and submitted to the Kolmogorov-Smirnov test, presenting a normal distribution.

After checking the normality of the results, the data were subjected to two-way ANOVA for repeated measures, considering the type of therapy as an independent factor and the categorical time the repetition factor, with the number of SM as the dependent variable. The level of statistical significance was 5% and the confidence interval was 0.95. Tukey ad-hoc tests were used for multiple comparisons.

## Results

Sixty-four children (37 girls and 27 boys) from the previous 90 enrolled in the study were able to attend all the recalls for saliva harvesting. The mean overall final age was 7.83. Group CHX was composed of 17 girls and 11 boys (29 total children), while the SDF group was composed of 20 girls and 16 boys (36 total children). The mean age of groups was 7.77 and 7.89 for CHX and SDF, respectively.

**Fig 1:**
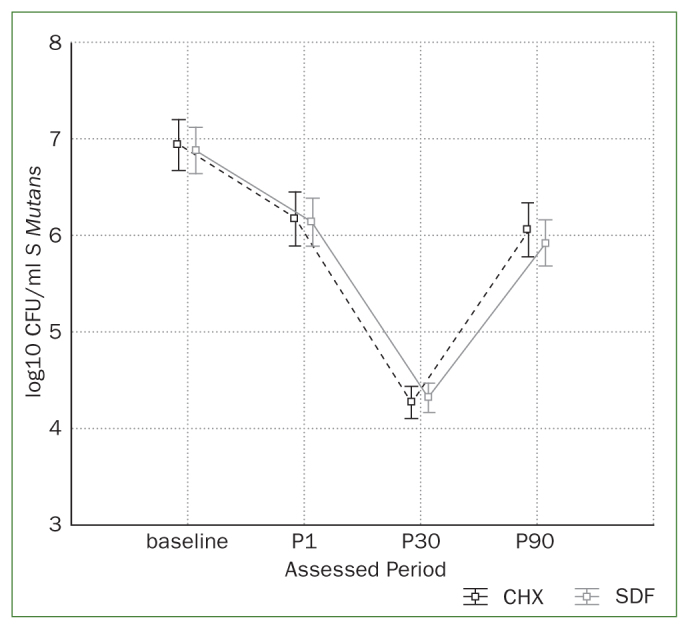
Graph with distribution of detected salivary *Streptococcus mutans* (log transformed CFU/ml and 0.95 confidence interval) according to the assessed periods.

CFU data for both groups at all assessed intervals are presented in Table 1. Log transformed data is presented in Figure 1.

**Table 1:** CFU data for CHX and SDF at all assessed periods

Assessed interval	Antimicrobial	
	CHX	SDF
Baseline	6.35 E+07 + 2.28 E+08^Aa^	8.79 E+07 + 4.62 E+08^Aa^
P1	4.16 E+06 + 6.60 E+06^Ba^	3.92 E+06 + 5.92 E+06^Ba^
P30	3.07 E+04 + 2.86 E+04^Ca^	3.06 E+04 + 3.09 E+04^Ca^
P90	2.92 E+06 + 3.48 E+06^Ba^	2.94 E+06 + 5.97 E+06^Ba^

Different capital letters show differences within columns (among different evaluation period for each antimicrobial). Different small letters show differences within lines (between antimicrobials for each evaluated period). Statistical analysis was performed with two-way repeated measures ANOVA using log transformed data.

Data were submitted to two-way repeated ANOVA and Tukey’s test after confirmation of normal distribution. CFU data were influenced by the assessing period (p <0.001), while no influence was detected for the type of antimicrobial used (p = 0.65) or the interaction of studied factors (p = 0.85).

The decrease in CFU/ml was similar for both antimicrobials. There was a statistically significant reduction at P1, with the greatest reduction at P30. At P90, CFU/ml SM levels had risen to the levels observed at P1.

The behaviour of both antimicrobials was similar for each assessed interval (Fig 1).

## Discussion

The motivation to perform the present study is related to the fact that dental caries is still highly prevalent in the population and other preventive approaches/products might aid the disease prevention, especially when considering underprivileged populations or specific groups of individuals within developed countries.^11,23^

*SM* was the chosen bacterium as it has been advocated in several studies as an indicator for dental caries risk/caries activity.^1,7,12,15^

The bacterial count over time was the main objective of the present research. During the initial assessment, the amount of SM was high, close to 70 times the minimal value for characterising individuals within the high caries risk group (above 1 × 10^6^ CFU/ml),^12,14^ a fact confirmed by the presence of at least one initial caries lesion per patient. Such observation supports the use of ICDAS criteria, once it allows for the diagnosis of incipient lesions and also the severity of cavitated lesions.^10^ The baseline assessment for inclusion of high caries risk children was important, as including participants with low bacterial counts would hinder the real preventive effect of the tested protocols. There is a debate in the literature about the actual number of CFU/ml of SM in saliva to characterise patients’ risk. The number varies from 105 to 10^6^ CFU/ml,^1,7,12,14^ with the discussion centring on the number of tooth and restorations present in the mouth. There is also a discussion of correlating real CFU/ml counts with scoring mechanisms present in clinically available products. The authors of this study decided to adopt the greater value (10^6^ CFU/ml), considering that initial caries lesions were clinically detected and that the included patients presented this bacterial counting level at the baseline.

After 24 h of antimicrobial application following the reported protocol, the levels of *S. mutans* CFU were reduced drastically (≅ 95%), being statistically significant. Such reduction is in agreement with studies reporting the bacterial effect of both antimicrobials.^8,18,22^ Contrary to a study reporting differences between 0.2% chlorhexidine solution and 38% SDF under an in vitro SM biofilm design,^22^ with better results for SDF, the present data resulted in a similar reduction for both antimicrobials after 24 h. These differences might be explained by the differences in concentration or presentation of antimicrobials, as the present study employed 1% CHX in a gel form, or by fact that the present study is a clinical study assessing salivary bacterial information. Two studies testing in vitro biofilm models^18,22^ resulted in an almost complete reduction of bacteria after the application of SDF. Such a fact is not observed in the present study once the salivary levels of SM were studied, suggesting biofilm models are more effective in determining the local activity of SDF considering the lesion microenvironment, a fact not observed when studying bacteria in saliva.

The greatest reduction in SM levels (≅ 99.95% in both groups) in the present study was observed at the 30-day assessment, with salivary levels of SM being lower than all other assessed periods. However, opposite to studies reporting complete reduction of SM,^18,22^ the salivary SM was not completely eliminated under the protocol tested. The present data also differs from studies assessing the bacterial effect of SDF using biofilm models,^18,22^ in which the reductions are observed during initial assessments. The possible explanation for this study’s 30-day findings is related to some aspects, such as the methodology employed for counting SM, the possible influence of the hardening of surfaces of teeth, and/or biofilm adherence. Differences in methodology are explained by the fact that biofilm bacteria might be directly affected by antimicrobials, as they are in close contact to the target area and their reduction is promptly perceived, a fact not detected while counting salivary bacteria. This last factor takes into account the whole oral condition/bacterial activity. The influence of the hardening of teeth and biofilm/SM adherence^21,25,30^ might also have influenced the results, as it would take time for both processes to reduce/control the levels of salivary SM. For the CHX group, the methodology discussion (biofilm vs salivary levels of bacteria) also helps to explain the similar lower results after 30 days. One should also consider CHX substantivity properties,^3^ in which this antimicrobial is reported to present an effect over time, possibly explaining the obtained results. Patient motivation and compliance to oral hygiene instructions might also have played a part. Changing diet and oral hygiene habits are known to be effective in changing the caries risk of patients. The authors are not able to state that such a situation happened in the present study, but are also not able to eliminate such a supposition.

After 90 days, a statistically significant increase was detected for both groups regarding the levels of salivary SM in comparison to the 30-day results. Despite such an increase, the values were still lower than the baseline data, but similar to the 24-h results. This fact reflects a possible reversal in the caries process and might be related to the diminishing effectiveness of the antimicrobials after the 24-h period, or to the patients not adhering to their daily oral hygiene. On the other hand, one should note the levels of salivary SM are still under the adopted range to classify patients as high caries risk,^12,14^ and as this was the last assessment of the study, the authors are not able to determine the real interval for reaching the salivary levels of SM correspondent to caries risk. According to the literature, SDF applications every 12 months are advocated, with clinical successes reported.^5,6^ Considering these successes and the multifactorial characteristics of the dental disease, one might assume other factors related to caries disease are being positively influenced, such as hardening of the dental structures and hindered biofilm adherence.^21,25,30^

Both antimicrobials presented a similar pattern of SM reduction within the tested periods (p = 0.65). This is an adequate scenario considering CHX is the gold standard of antimicrobials in controlling/paralysing caries disease,^15,16^ and SDF presents some advantages against CHX. SDF properties such as hardening of dental structures^21,25^ by forming fluorohydroxyapatite,^19^ hindering biofilm adherence,^30^ and low cost^9^ support its indication for caries arresting/prevention.

Despite the reported advantages of SDF, it does have a staining property. Silver iodine, a white substance sensitive to light, is formed when using SDF and it is responsible for tooth staining.^30^ A strategy for avoiding tooth staining is based on applying a solution of potassium iodine after SDF, with no influences on SDF efficiency, has been reported.^13^ Potassium iodine was used in the present study and tooth staining was not detected within the participants.

Understating the influence of oral hygiene, diet and the socioeconomical status of the patients on the outcome of the present study would be beneficial. However, by subdividing the patients according to such parameters would have led to a decreased statistical power and the results might have been biased, as the study was not designed for such analysis and, also, the drop-out rate was considerable. Moreover, the study being designed as a randomised clinical trial, the authors decided to classify it as exploratory due to the non-retention of participants and the uneven distribution by groups, although the number of analysed participants was still within the number obtained in the sample size calculation.

Considering all the reported information on the presented data, SDF presented a similar 3-month antibacterial activity to CHX in high caries risk children. Based on its advantages regarding tooth surface hardening, hindered biofilm adhesion and low cost, it should be indicated in caries control.

## Conclusion

SDF presented a similar antibacterial effect as CHX for a period of 90 days. At that period, bacterial levels increased, but did not reach the baseline levels. Such a result endorses its clinical use as an adjunct therapy for dental caries control.
